# Autonomous mission planning for planetary surface exploration using a team of micro rovers

**DOI:** 10.3389/frobt.2025.1565173

**Published:** 2025-04-09

**Authors:** Sarah Swinton, Jan-Hendrik Ewers, Euan McGookin, David Anderson, Douglas Thomson

**Affiliations:** James Watt School of Engineering, University of Glasgow, Glasgow, United Kingdom

**Keywords:** planetary exploration, robot team, coordination, autonomy, mission planning, micro rover

## Abstract

One of the fundamental limiting factors in planetary exploration is the level of autonomy achieved by planetary exploration rovers. This study proposes a novel methodology for the coordination of an autonomous multi-robot team that evaluates efficient exploration routes in Jezero crater, Mars. A map is generated consisting of a 3D terrain model, traversability analysis, and probability distribution map of points of scientific interest. A three-stage mission planner generates an efficient team-wide route, which maximises the accumulated probability of identifying points of interest. A 4D RRT* algorithm is used to determine smooth and flat paths for individual rovers, following the team-wide route planner, and prioritized planning is used to coordinate a safe set of individual paths. The above methodology is shown to coordinate safe and efficient rover paths, which ensure the rovers remain within their nominal pitch and roll limits throughout operation.

## 1 Introduction

Multi-robot teams are widely used to carry out tasks that are too complex, too risky, or too time-consuming for a single robot to complete. This is often achieved by leveraging the capabilities of heterogenous robots, with individual specialisations (Parker, 1998), allowing a wider array of tasks to be carried out at any given time (Gautam and Mohan, 2012). Alternatively, a team of homogeneous robots may cooperate to complete tasks more efficiently than a single robot (Rogers et al., 2013). These beneficial characteristics of multi-robot teams have seen them used in applications including firefighting (Roldán-Gómez et al., 2021), search and rescue (Worrall, 2008), and planetary exploration (Farley et al., 2020).

NASA’s Mars 2020 Mission has been the first planetary exploration mission to utilise two robotic platforms working in close proximity: the Perseverance Rover, and the Ingenuity Mars Helicopter (Farley et al., 2020). Further research has investigated the efficacy of multi-robot teams for planetary exploration on both the surface (Fong et al., 2021; Swinton and McGookin, 2022) and in caves (Fink et al., 2015; Vaquero et al., 2018). Planetary exploration missions present an opportunity to capitalise on the beneficial characteristics of robot teams outlined above. A team of cooperative rovers would have a significantly increased sensor footprint, and therefore allow larger regions to be investigated, compared to current single rover missions. The team of rovers could complete their own separate tasks, contributing to an overall mission goal, or work collaboratively to carry out a larger central goal, e.g., to map an area.

Despite the advantages outlined above, multi-robot planetary exploration presents several challenges. Planetary exploration rovers (PERs) operate in extremely remote and hazardous environments, where collisions could lead to the loss of the rovers involved and result in severe degradation to the group’s data collection capabilities. The paths of each member of the rover team must, therefore, be coordinated such that no collisions occur between them as they traverse paths towards their respective targets. This coordination cannot be carried out by human operators, due to the tele-operation constraints present in planetary exploration missions. Low-level pre-planning of rover team operations by human operators is also impractical, due to increased complexity of the entire system compared to a single robot. As system complexity increases, so too does the workload and stress of a human operator. Therefore, high levels of autonomy within multi-robot systems are essential to reduce the required cognitive load on human operators (St-Onge et al., 2019).

Methodologies for autonomous multi-robot coordination have been extensively explored in the academic literature. The selection of an appropriate coordination strategy is highly dependent on the specific mission requirements of the multi-robot system (MRS). One of the simplest and most widely employed strategies, particularly in teams of unmanned ground vehicles (UGVs), is the leader-follower formation (Abujabal et al., 2023). This approach follows a centralized paradigm in which a designated “leader” robot possesses complete knowledge of waypoints and target locations, while “follower” robots rely entirely on the leader for path planning and navigation (Liu and Bucknall, 2018). Despite its ease of implementation, the leader-follower approach presents several critical limitations. Notably, follower robots lack independent path-planning capabilities, which may compromise mission robustness, particularly in high-risk applications such as planetary exploration. In such environments, dependence on a single leader introduces a potential single point of failure, necessitating additional mechanisms to enhance resilience and mitigate the risks associated with centralized control. Furthermore, strict formation constraints may be unsuitable for highly unstructured or dynamic environments.

To address these challenges, a decentralised approach to MRS path planning can be taken. Within a decentralised framework, each robot has the ability to plan and follow its own path. One such approach allows robots to follow independent paths before regrouping at predefined waypoints. This method, which can be facilitated using Rapidly-exploring Random Trees (RRT) for efficient navigation through cluttered environments, provides a more adaptable and robust coordination framework (Liu et al., 2014). However, operating in dynamic environments is inherent in the nature of MRS. Therefore, it is crucial that any paths generated by members of the team be coordinated such that no collisions occur. Prioritised planning is a decoupled approach to the path planning for MRS (Erdmann and Lozano-Perez, 1987). This approach has two key stages. First, initial robot paths are planned. Next, the algorithm seeks to resolve any conflicts between paths, replacing individual paths until a set of collision free paths is obtained. Prioritised planning has been shown to outperform fixed path coordination, whereby the velocity profiles of individual robots are adjusted to avoid collisions, by eliminating dynamic collisions in teams of micro-rovers for planetary exploration (Swinton and McGookin, 2022). However, prioritised planning faces a challenge: the computational requirements increase with the number of robots and the map size, negatively impacting scalability (Heselden and Das, 2023). Therefore, prioritised planning is most appropriate for MRS with a small number of robots.

This work addresses the issue of mission planning for multi-robot planetary exploration, by proposing a mission planner that enables a team of five micro-rovers to autonomously explore a large region of the surface of Jezero Crater, Mars. Using this approach, human operators need only provide a partial mission map. The micro-rovers examined in this study are designed to be low-cost. Consequently, while not the primary focus of this work, each micro-rover may possess unique roles and hardware capabilities. This variability makes independent travel to separate points of interest impractical, as team members must remain in proximity to one another to provide necessary capabilities when required, or to assist in the event of faults and failures. The exploration approach within this work therefore allows rovers to spread out while following a team-wide route. The key contributions of the work are:

• a mapping approach for planetary exploration mission sites that combines data on terrain traversability and the location of points of scientific interest using probability distribution maps
• a clustered exploration approach for a team of low-cost micro-rovers in a 3D environment utilising RRT* and prioritised planning.

This paper is set out in the following manner. Section 2 sets out the multi-rover system. Section 3 defines the mission planning approach. Section 4 describes the experiments carried out to evaluate the proposed methods. Finally, Section 5 summarises the outcomes from this study and the overall conclusions that can be drawn from this work.

## 2 Modelling a team of suitable micro-rovers

### 2.1 Selecting a suitable micro-rover platform

Planetary exploration missions are subject to strict financial and payload constraints (Planetary-Society, 2024). It would not, therefore, be feasible to plan a multi-rover mission where each individual rover has the technological capabilities of, for example, the Perseverance rover, and in turn the same constraints. A multi-rover team should be able to be stowed in a launch capsule that has regularly been used to hold a single, larger rover. Further, a reduced sensor suite may have to be employed. However, the members of a multi-rover team could be given specialised roles, each of which has a distinct and complementary set of sensors, to allow the team to carry out complex exploration tasks in a similar way to existing single rover systems. These specialised roles could include scouts, drillers, image analysers, and sample storers/carriers. It is important to note that reduced individual capability should not come at the cost of overall mobility as PERs must be able to traverse uneven terrain and slopes.

**FIGURE 1:**
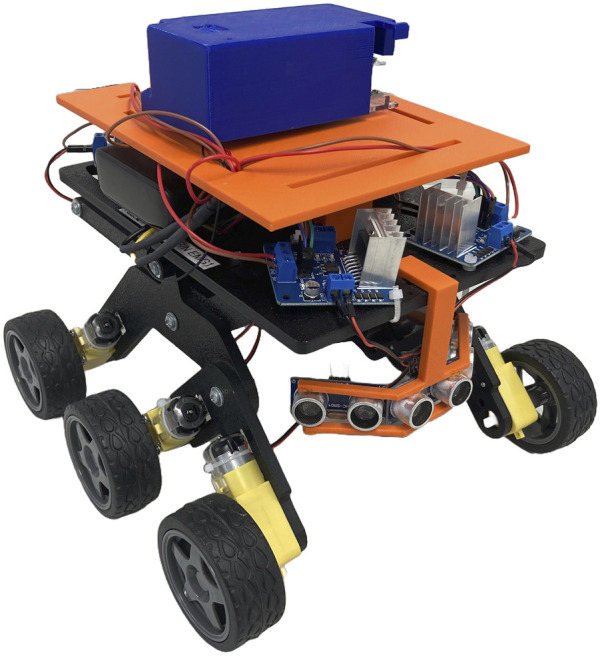
Prototype of the micro-rover, developed at the University of Glasgow, using the Servocity chassis and COTS components.

To satisfy these requirements, a prototype micro-rover has been developed at the University of Glasgow as a suitable analogue for this work (Figure 1). The chassis of this micro-rover is a rocker-bogie runt (ServoCity, 2024). The prototype has been designed using commercial-off-the-shelf (COTS) components. The micro-rover has a small form factor (0.271 m ×0.251 m ×0.144 m), and a six-wheel rocker bogie suspension in line with the baseline mobility characteristics of current PERs (Flessa et al., 2014). The multi-rover team consists of five RBR rovers, which have been simulated using MATLAB.

### 2.2 Mathematical modelling

**FIGURE 2:**
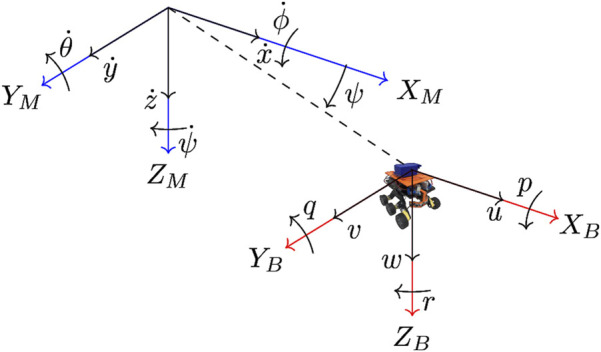
Mars-fixed (X_M_, Y_M_, Z_M_ (blue)) and rover body-fixed axes (X_B_, Y_B_, Z_B_ (red)) for the modelled rover (Swinton et al., 2024b).

Central to the implementation of an robust simulation of each micro-rover is the development of an accurate mathematical model, which considers both the dynamics and kinematics of the rover. For this mathematical model, two reference frames are defined: the Mars-fixed frame, and the rover body frame. The Mars-fixed frame has an inertially fixed origin, and the rover body frame has its origin at the rover’s centre of gravity. These axes are oriented following the North East Down (NED) system, where positive Z motion is directed downwards from the rover’s centre of gravity (Fossen, 1994), and are shown in Figure 2.

Here, the rover’s translational velocities are u , v , and w . The rover’s rotational velocities are p , q , and r . The rover’s rigid body dynamics, with reference to the body-fixed frame and Mars-fixed frame, can be described by the matrix relationships shown in Equations 1–3 (Fossen, 1994).

$$\left[\begin{array}{c} \dot{v}\\ \dot{\eta } \end{array}\right]=\left[\begin{array}{cc} \alpha \left(v\right) & \beta \left(v\right)\\ J\left(\eta \right) & 0 \end{array}\right]\left[\begin{array}{c} v\\ \eta \end{array}\right]+\left[\begin{array}{c} -M^{-1}\\ 0 \end{array}\right]\tau$$ (1)

$$\alpha \left(v\right)=-\left(C\left(v\right)+D\left(v\right)\right)M^{-1}$$ (2)

$$\beta \left(v\right)=-g\left(v\right)M^{-1}$$ (3)

Here, v is the body-fixed velocity vector and η is the inertially fixed position/orientation vector. M is the mass and inertia matrix, C(v) is the Coriolis matrix, D(v) is the damping matrix, g(v) represents the gravitational forces and moments, J(η) is an Euler matrix representing the kinematic transformation from the body-fixed reference frame to the Mars-fixed reference frame, and the τ vector represents the forces and moments generated by the actuators. These result in the equations of motion shown in Equation 4.

$$\begin{array}{cc} X & =m\left(\dot{u}+w.q-v.r\right)\\ Y & =m\left(\dot{v}+u.r-w.p\right)\\ Z & =m\left(\dot{w}+v.p-u.q\right)\\ K & =J_{x}.\dot{p}+\left(J_{z}-J_{y}\right).q.r\\ M & =J_{y}.\dot{q}+\left(J_{x}-J_{z}\right).p.r\\ N & =J_{z}.\dot{r}+\left(J_{y}-J_{x}\right).p.q \end{array}$$ (4)

Here, X , Y , and Z are the rover’s surge, sway, and yaw forces. The rover’s roll, pitch, and yaw moments are represented by K , M , and N , respectively. The rover’s mass is represented by m , its moments of inertia about the x, y, and z-axes are represented by $J_{x}$ , $J_{y}$ , and $J_{z}$ , respectively.

### 2.3 Guidance and control systems

**FIGURE 3:**
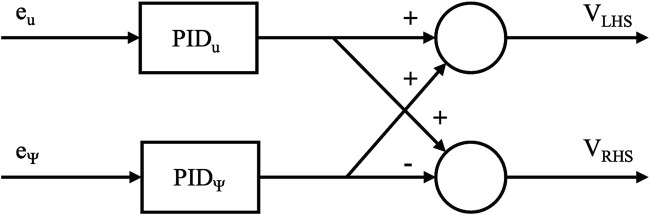
Architecture of the micro-rover’s control system, providing voltages to the left and right hand side motors.

Each simulated rover is equipped with a guidance and control system. A line-of-sight algorithm (Breivik, 2003) is used to enable the rover to navigate towards a waypoint. Path following can then be carried out by iterating through a series of waypoints. The control system consists of two PID controllers, as shown in Figure 3. The first of these PID controllers corresponds to the rover’s surge velocity, u , by evaluating the difference between the desired and measured surge velocity values, $e_{u}$ . As the RBR does not have steerable wheels, skid steering is used. Using this method, a difference between the voltages supplied to the left hand side (LHS) motors, $V_{\mathrm{LHS}}$ , and right hand side (RHS) motors, $V_{\mathrm{LHS}}$ , motors will result in turning motion. The second PID controller therefore utilises the difference, $e_{\psi }$ , between the rover’s desired and measured heading, to provide a control signal that allows the rover to turn.

## 3 Autonomous mission planner

### 3.1 Mission planner architecture

**FIGURE 4:**
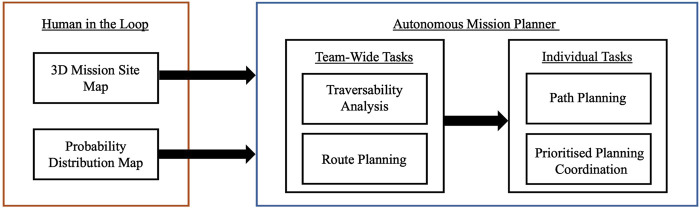
High-level architecture of the autonomous mission planner, encapsulating the role of a human operator, as well as both team-wide and individual autonomous tasks.

In order to fully leverage the capabilities of a rover team, the role of a human operator must be carefully considered. Previous work has shown that it is not desirable to have a human operator attempt to fully manage more than one robot (St-Onge et al., 2019). The autonomous mission planner proposed within this work allows a human operator to specify the mission site to be explored, and provide a probability distribution map that defines which sections of the map are likely to contain points of scientific interest. Using these inputs, the autonomous mission planner will identify an efficient route - which maximises the likelihood of identifying points of scientific interest, while avoiding high risk terrain regions. Once a team-wide route has been established, individual rover paths are identified. Each individual path should avoid overlap with the paths of team mates, in order to increase the overall sensor footprint of the team. To ensure each path will not incur collisions, prioritised planning is utilised. This architecture is set out in Figure 4, and is discussed throughout the remainder of this section.

### 3.2 3D mission site map

**FIGURE 5:**
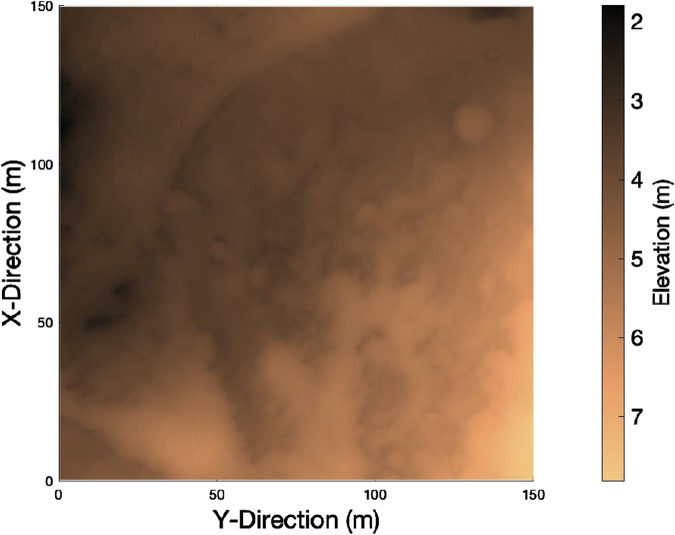
3D mission site map of the selected region of Jezero Crater, Mars.

In 2006, the Mars Reconnaissance Orbiter began operation surveying the surface of Mars (Zurek and Smrekar, 2007). One of the primary instruments on the orbiter is the High-Resolution Imaging Science Experiment (HiRISE). HiRISE captures images of the surface of Mars with resolution of ∼30 cm per pixel (from an altitude of 300 km) (Arizona-HiRISE, 2024), providing terrain information that is sufficient for high-level mission planning. Using HiRISE data, a 3D mission site map of areas on the surface of Mars can be generated as a matrix of latitude, longitude, and elevation points. Human operators can, therefore, provide the rover team with a map of their environment, i.e., the area that is to be searched. For this work, a 1500 m ×1500 m mission site has been selected from within the Jezero crater. As the rover used in this work is approx. 1/10 th the size of the Perseverance rover, the environment model is scaled to 150 m ×150 m. Within the simulation, the Martian surface is composed of a 600×600 block grid, where each block is 0.25 m ×0.25 m. Figure 5 shows the 3D terrain model that has been simulated in MATLAB.

### 3.3 Probability distribution map

**FIGURE 6:**
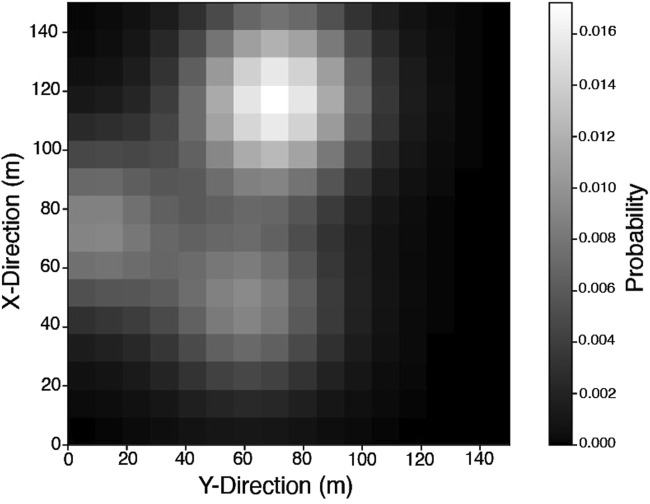
Representative example of a PDM, $p\left(\vec{x}\right)$ , that covers the 3D mission site map.

The second input to the autonomous mission planner is a probability distribution map (PDM), that defines the probability of finding a point of interest (POI) at any given position in a continuous manner (Figure 6). What constitutes a POI varies with the scientific goals of a given planetary exploration mission. Selection of such scientific objectives has been considered out with the scope of this study. Within this work, PDMs are modelled which cover the entire map, and contain small regions of high probability.

These PDMs modelled as the sum of G bivariate Gaussians (Lin and Goodrich, 2014) such that a point on map at coordinate $\vec{x}\in R^{2}$ has a probability of containing the POI, $p\left(\vec{x}\right)$ , as shown in Equation 5.

$$p\left(\vec{x}\right)=\frac{1}{G}\overset{G}{\underset{i=0}{\sum }}\frac{\exp\left[-\frac{1}{2}{\left(\vec{x}-{\vec{\mu }}_{i}\right)}^{T}{\vec{\sigma }}_{i}^{-1}\left(\vec{x}-{\vec{\mu }}_{i}\right)\right]}{\sqrt{4\pi^{2}\det{\vec{\sigma }}_{i}}}$$ (5)

Here ${\vec{\mu }}_{i}$ and ${\vec{\sigma }}_{i}$ are the mean location and covariance matrix of the i th bivariate Gaussian respectively. For the purpose of this study, these values are randomly generated with G=4 . In a real-world scenario, this can be generated using an algorithm such as J1 for wilderness search and rescue (Ewers et al., 2023).

### 3.4 Traversability analysis

**FIGURE 7:**
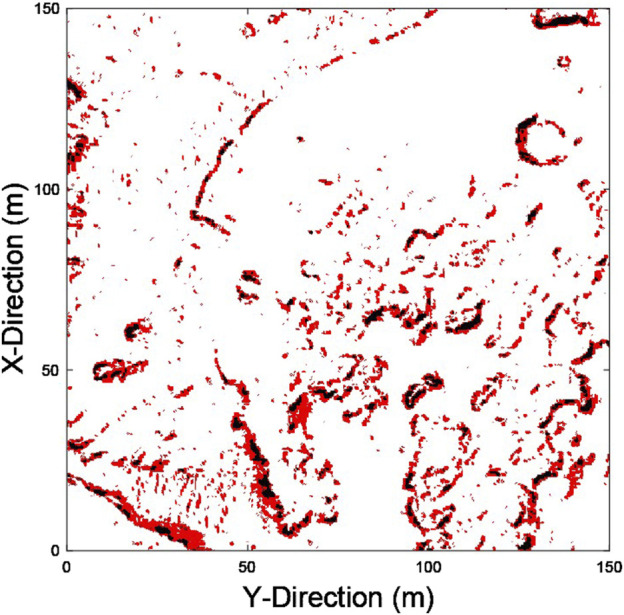
Traversability analysis of the selected mission site. Traversable terrain is shown in white, high risk terrain is shown in red, and impassable terrain is shown in black.

Planetary exploration rovers are subject to slip as they traverse steep slopes and rough terrain. To reduce the number of potential collisions due to slip, smooth and flat terrain should be explored when possible. A traversability analysis is carried out on the 3D mission site map where-by the elevation of neighbouring blocks in the 600×600 block grid are compared to find the slope angle required to traverse between the blocks. A given block inherits the worst-case slope angle. Nominal pitch and roll limits of 15° , in line with the nominal operational limits of the Perseverance rover (Rankin et al., 2020), have been implemented. The traversability analysis determines which regions of each map are traversable, high risk, and impassable. Traversable terrain is safe to explore. High-risk terrain has a slope of θ>=10° . Impassable terrain has a slope of θ>=15° . Figure 7 shows the resulting traversability map for the selected mission site. Here 89.50% of the map is traversable, 8.36% of the map is high risk, and 2.14% of the map is impassable.

### 3.5 Team-wide route planning

PDM-based search planning differs from classical A to B path planning in that it aims to accumulate the maximum probability, $p\left(\vec{x}\right)$ , along a path. The search planning algorithm selected for this work is LHC_GW_CONV (Lin and Goodrich, 2009). LHC_GW_CONV approaches the path planning problem by segmenting the search area into N×M cells, such that each cell is as large as the search footprint. In this study, the search footprint has a diameter of 5 m (i.e., each of the five rovers in the team has a 1 m search diameter).

LHC_GW_CONV is based on the Local Hill Climbing (LHC) optimisation method, which considers all eight cells around the current position and selects the cell with the highest probability value. This cell is selected for the next step and the previous cell is marked as having been completely seen with a value of 0. This does not prohibit future traversing of this cell, but strongly discourages it to mitigate the risk of deadlock, which could necessitate premature termination. In the case that multiple cells have equal values, a 3×3 normalized box blur convolution kernel, ω , is applied to each equally valued cell (Equation 6).

$$\omega =\frac{1}{9}\left[\begin{array}{ccc} 1 & 1 & 1\\ 1 & 1 & 1\\ 1 & 1 & 1 \end{array}\right]$$ (6)

The convoluted value $p^{\mathrm{conv}}\left(\vec{x}\right)$ for any position is found using Equation 7.

$$p^{\mathrm{conv}}\left(\vec{x}\right)=\omega *p\left(\vec{x}\right)=\overset{1}{\underset{i=-1}{\sum }}\overset{1}{\underset{j=-1}{\sum }}\omega \left(i,j\right)p\left(\vec{x}-\left[\begin{array}{c} i\\ j \end{array}\right]\right)$$ (7)

The cell with the largest vale of $p^{\mathrm{conv}}\left(\vec{x}\right)$ is selected for the next step. Without any further modifications, the algorithm would fully explore the nearest local maxima fully before considering others. This is a common problem with LHC. To encourage exploring the entire PDM, the concept of global warming, GW, is introduced. Here, a value C is subtracted from the PDM a l number of times, where $C=p_{\max}/l$ and $p_{\max}$ is the global maxima. The PDM is then updated through Equation 8.

$$p^{\prime }\left(\vec{x}\right)=\left\{\begin{array}{cc} p\left(\vec{x}\right)-C,\; & p\left(\vec{x}\right)>C\\ 0,\; & else \end{array}\right.$$ (8)

After all l GW steps are completed, each path is evaluated against the original PDM $p\left(\vec{x}\right)$ and the one with the maximum accumulated probability is returned.

### 3.6 Identifying and coordinating individual rover paths

Once a team-wide route has been established, individual rover paths can be evaluated. Each of these individual paths will follow the team-wide route, whilst avoiding collisions and increasing the sensor footprint of the team. This increased sensor footprint (compared to having the rovers follow behind one another at a safe distance) is a result of both the random nature of the RRT* algorithm, and the collision avoidance implemented within the coordination algorithm. In this work, an RRT* path planning algorithm is used to identify individual rover paths. First set out by LaValle, a Rapidly-exploring Random Tree (RRT) is a randomised data structure that facilitates path planning for non-holonomic vehicles (LaValle, 1998). Karaman and Frazzoli introduced RRT*, an asymptotically optimal extension of RRT; as the number of nodes on the tree increases, the cost of the returned solution converges on an optimal region (Karaman and Frazzoli, 2011).

In the general form of RRT*, path cost is based purely on distance. However, for robots in 3D environments with varying terrain, the shortest path may not always be the preferred path. For planetary exploration robots, paths which are longer, but smoother and flatter are often preferable for robot safety. Equation 9 shows the cost function implemented in order to produce paths which are obstacle-free, smooth, and flat (Takemura and Ishigami, 2017). Four cost components are utilised: path length (R) , roll (ϕ) , pitch (θ) , and required turning angle from the previous node to current node (Δψ) .

$$cost\left(q_{i}\right)=W_{R}\frac{R_{i}}{N_{R}}+W_{\phi }\frac{\phi_{i}}{N_{\phi }}+W_{\theta }\frac{\theta_{i}}{N_{\theta }}+W_{\psi }\frac{\Delta \psi_{i}}{N_{\psi }}$$ (9)

In the above equation, $q_{i}$ is the node currently being checked, W represents a weighting factor for each cost component such that the weights sum to 1. The weights $W_{\phi }$ and $W_{\theta }$ are set to 0.4 with $W_{R}$ and $W_{\psi }$ set to 0.1 such that the flatness of paths is prioritised. Rovers are prone to slip when navigating slopes, which can lead to localization errors since wheel encoders do not capture this movement. Severe cases of slip can cause a rover to deviate significantly from its intended path, potentially resulting in collisions with teammates. In this study, where rovers operate in close proximity, slip-risk is reduced by prioritising path flatness. N represents a normalisation factor to make each index dimensionless. These values are based on the maximum valid value of the respective cost components (i.e., the maximum step the RRT can take is 1 m, hence $N_{R}$ = 1 m). The respective normalisation factors are $N_{R}$ = 1 m, $N_{\phi }$ = 15° , $N_{\theta }$ = 15° , and $N_{\psi }$ = 60° .

For any given pair of start and target points, boundaries are set to ensure the RRT* path planner searches only a small, relevant chunk of the full mission site (i.e., 2 m clearance of the start and target points in both the X and Y directions). These boundaries ensure the scalability of this methodology to larger mission sites as, regardless of size of the mission area, the RRT* algorithm will only be required to search over one path segment at a time. The RRT* planner searches the bounded area by growing the tree until the maximum number of nodes has been reached. The maximum number of nodes selected in this work is 1,250, which is sufficient to thoroughly search the bounded area, but does not incur a high run time.

**FIGURE 8:**
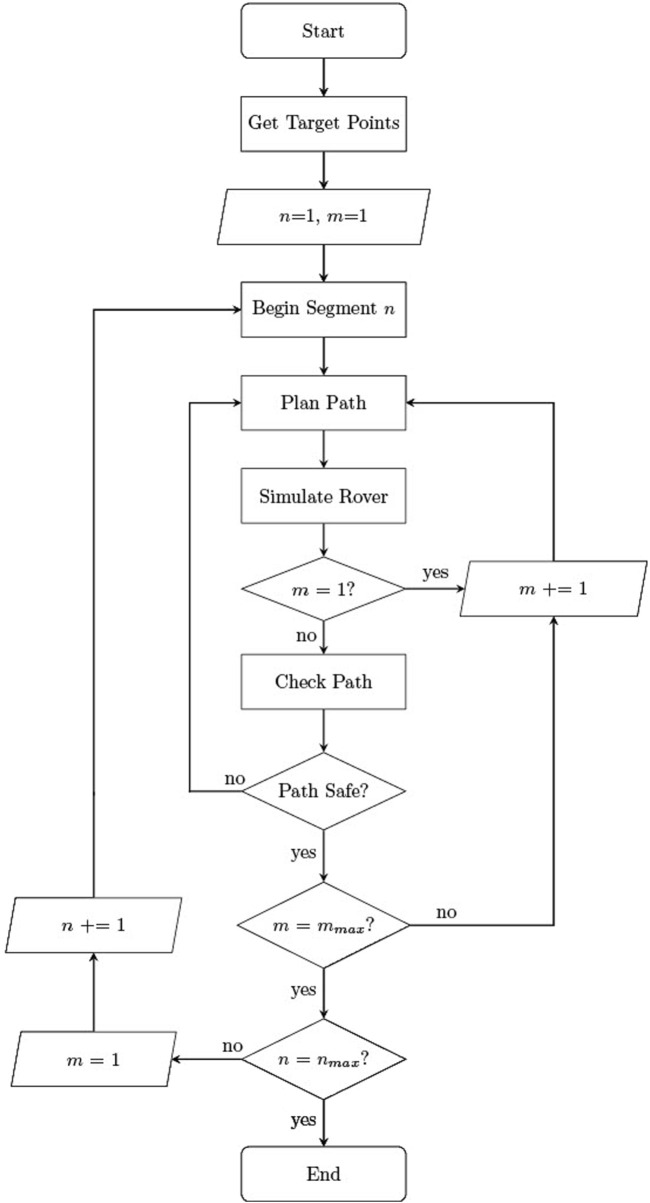
Block diagram of prioritised planning coordination algorithm, where n represents the index of the path segment, and m represents the index of the rover team member.

The paths generated for each rover must be coordinated such that no collisions occur. For this purpose, prioritised planning is utilised. Prioritised planning is a 4D coordination methodology, which has been shown to eliminate dynamic collisions under nominal conditions (i.e., when no faults are present in the system), outperforming other common coordination algorithms such as fixed path coordination (Swinton and McGookin, 2022). Using this method, an initial set of safe paths can be coordinated offline. Each rover is awarded a priority index. The highest priority rover’s path is planned first, and a simulation is run to acquire 4D positional and temporal data as the rover traverses its planned path. The algorithm then attempts to plan a path for the second rover, comparing the positions of both rovers at each time-step to check for potential collisions. If collisions are detected, another path planning attempt is made for the second rover. This process repeats until the second rover’s path is deemed ‘safe’. The algorithm then attempts plan a collision free path for each subsequent rover, descending in priority. This process is carried out sequentially for each segment of the team-wide route. This process is outlined in Figure 8, where n is the segment index (with a maximum value of n_max_), and m is the rover index. The maximum rover index is $m_{\max}$ , which has a value of 5 in this case.

## 4 Results

### 4.1 Simulation set-up

To evaluate the ability of the multi-layer mapping and mission planning methodologies to generate safe and efficient paths autonomously, various test scenarios have been considered within the Jezero crater mission site. Throughout this section, a representative scenario has been illustrated where appropriate. In each test scenario, the PDM is varied randomly. This results in unique target generation for each test case, and consequently different areas of the mission site are explored. The experimental results are set out as follows. First, the generation of team-wide route plans is described. Second, the safety of the coordinated paths is assessed. Finally, the efficiency of autonomous exploration plan is analysed.

### 4.2 Generation of team-wide route plan

**FIGURE 9:**
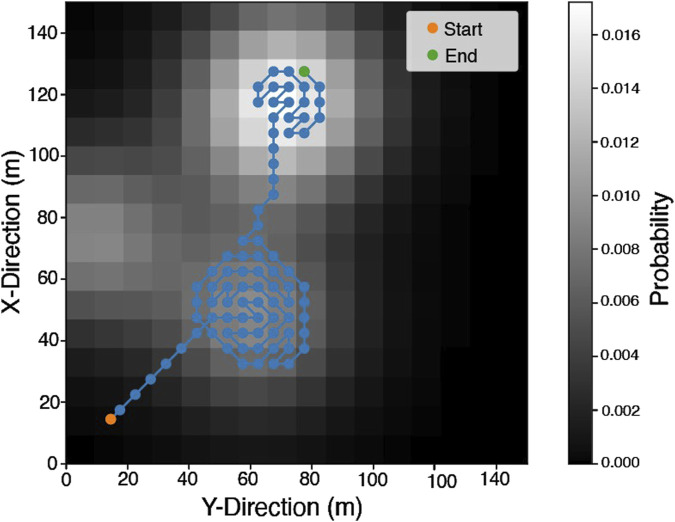
A set of target points generated using a random PDM (p(x)) and LHC_GW_CONV.

In each mission scenario, a random PDM is generated, and a team-wide route plan is evaluated using LHC_GW_CONV. This route plan consists of a set of target points, which maximise the accumulated probability of capturing the POI along the path. Figure 9 shows the set of target points generated for a representative scenario. In the case shown, which features overlapping regions of high probability density, it can be seen that LHC_GW_CONVguides the mission planner away from searching only one local maxima. While the representative PDM example shown in Figures 6, 9, 11 includes overlapping exploration areas, the extent of these overlaps varied across the full test set. In cases without overlapping regions of high probability, LHC_GW_CONVprovided paths which would explore the initial region fully, before moving to the next.

### 4.3 Generation of safe and efficient rover paths

Throughout the test set, data has been sampled from each rover at a frequency of 10 Hz, resulting in a total of 942,321 samples for the rover poses. Within the resulting data set, rover pitch and roll measurements exceeded the nominal threshold of 15° on only 25 occasions. This means that the RRT* path planner has been able to provide paths that keep the rovers within their nominal pitch and roll limits for 99.99% of operation. The few instances where pitch or roll exceed nominal limits are due to slip as the rover attempts to traverse waypoints, causing it to veer slightly off the ideal trajectory.

**FIGURE 10:**
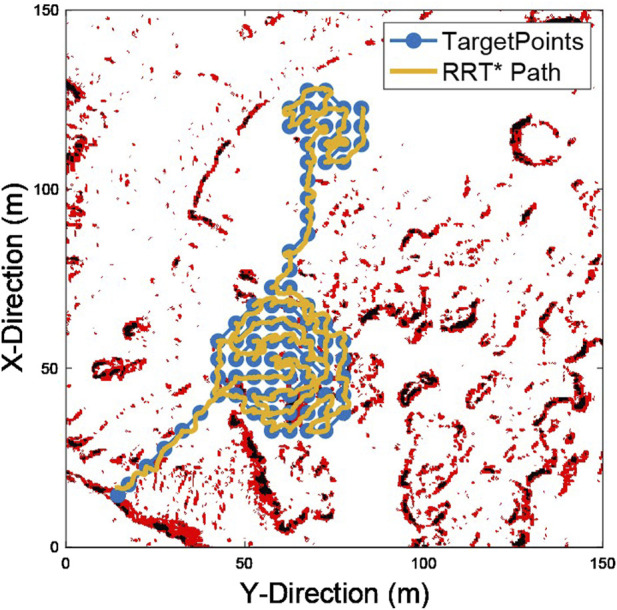
A representative example of a path generated by RRT* to explore the team-wide route.

For each pair of sequential targets within the team-wide route plan, the RRT* path planner attempts to find a safe path using the traversability map. Figure 10 shows a path generated over a full set of target points. A single rover path is shown for clarity.

**FIGURE 11:**
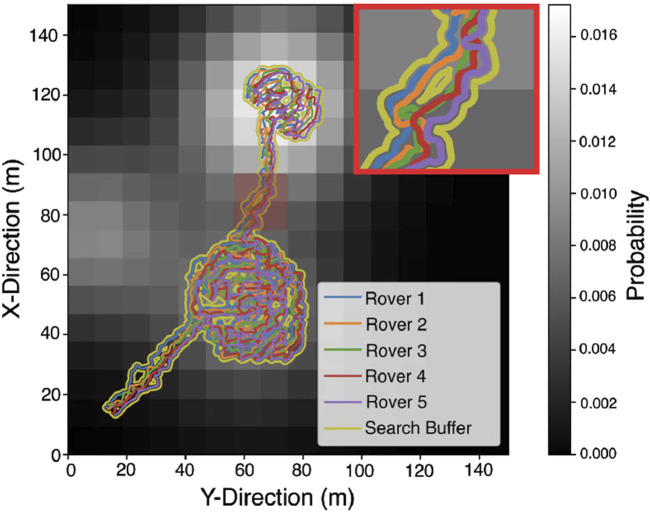
A representative example of fully coordinated rover team trajectories over a full mission scenario. An expanded view of a subset of the paths (highlighted in red) is shown in the top right corner of the figure.

The prioritised planning coordination algorithm invalidates any RRT* path planning attempts that cause a rover to collide with higher priority team mates. As such, the selected method generates safe paths in each mission scenario. Figure 11 shows an example of the full trajectories of five rovers in a test case. The boundaries of the team’s sensor footprint are shown by the search buffer. Gaps in the search buffer can be observed where the rovers follow paths around impassable terrain.

### 4.4 Exploration efficiency

The full mission site covers ${22500\text{m}}^{2}$ . Considering a team of five rovers, each with a sensor footprint of 1 m radius, each rover would be required to travel ∼4500 m for the team to acquire an accumulated probability of locating the POI approaching 1.

**FIGURE 12:**
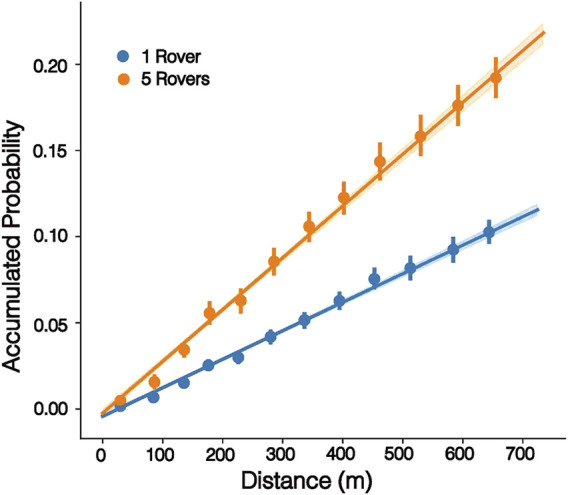
Accumulated probability over distance travelled for a single rover compared to a team of five rovers.

Over the mission scenarios considered, the average time taken for a rover team to traverse the 64 target points was 2,295.25 s (38.26 min), with an average trajectory of 653.68 m. By utilising the proposed autonomous exploration method, the rovers are able to acquire almost p(x) = 0.2 while travelling only 650 m. Figure 12 shows the increase in accumulated probability for a single rover, compared to that of a team of five rovers.

These results show that the proposed methodologies allow a large area to be searched effectively by a small team of rovers, generating paths which increase accumulated probability and can be carried out in less than one sol of operation.

## 5 Conclusion

This work tackled the problem of autonomous exploration for multi-rover teams by considering a mapping approach to identify high-interest, safe, regions of terrain, and a novel mission planning methodology that allows the rover team to safely and efficiently explore a given mission region. The mapping approach in this work was composed of three stages: a 3D digital terrain model of Jezero crater (generated using HiRISE orbiter data), a traversability analysis of the mission site, and a PDM which maps the areas of the mission site most likely to contain scientific POI. The mission planning methodology consisted of three stages. First, PDM-based search planning was used to identify a team-wide route plan which accumulates the highest probability of finding scientific POI. Second, a 4D RRT* path planner was used to identify flat, smooth paths between targets. Finally, prioritised planning coordination was used to ensure that each rover path was safe, i.e., did not incur any collisions.

While this study focuses on a small team of micro-rovers consisting of only five members, it is important to acknowledge that the proposed prioritized planning algorithm may not be inherently scalable to larger robotic teams (Heselden and Das, 2023). Addressing this limitation requires further investigation into the algorithm’s applicability across alternative multi-robot configurations. A potential approach to improving scalability involves the hierarchical organization of larger teams, where micro-rovers are assigned distinct roles based on their capabilities. In this framework, a larger team could be partitioned into multiple sub-teams, each possessing a complete set of functional capabilities. These sub-teams could then operate semi-independently, investigating distinct points of interest (POIs) while maintaining coordinated mission objectives. Future work should explore the feasibility of such an approach, assessing its impact on efficiency, robustness, and overall mission performance.

Additionally, the coordination algorithm proposed in this work does not impose penalties for re-exploring previously visited areas of the map. Instead, coverage naturally emerges from the inherent randomness of the RRT* algorithm and the collision avoidance mechanisms integrated into the coordination framework. Future research should focus on optimizing the degree of exploration overlap among team members to enhance the overall efficiency of the proposed clustered exploration algorithm.

The performance of the proposed methods has been evaluated over a set of randomly generated PDMs in the mission site of Jezero crater, Mars. It has been shown that collision-free autonomous exploration can be carried out efficiently over an area of ${22500\text{m}}^{2}$ in an average time of 38.26 min. Further, the rover trajectories generated during testing had an average length of 653.68 m; comparable to the current record for longest distance driven without human review by a planetary exploration rover. Therefore, the approach proposed in this paper successfully enables safe and efficient autonomous exploration of a 3D environment using a team of planetary exploration rovers.

## Data Availability

The raw data supporting the conclusions of this article will be made available by the authors, without undue reservation.
